# Mjolnir: Extending HAMMER Using a Diffusion Transformation Model and Histogram Equalization for Deformable Image Registration

**DOI:** 10.1155/2009/281615

**Published:** 2009-08-11

**Authors:** Lotta M. Ellingsen, Jerry L. Prince

**Affiliations:** Department of Electrical and Computer Engineering, The Johns Hopkins University, Baltimore, MD 21218, USA

## Abstract

Image registration is a crucial step in many medical image analysis procedures such as image fusion, surgical planning, segmentation and labeling, and shape comparison in population or longitudinal studies. A new approach to volumetric intersubject deformable image registration is presented. The method, called Mjolnir, is an extension of the highly successful method HAMMER. New image features in order to better localize points of correspondence between the two images are introduced as well as a novel approach to generate a dense displacement field based upon the weighted diffusion of automatically derived feature correspondences. An extensive validation of the algorithm was performed on T1-weighted SPGR MR brain images from the NIREP evaluation database. The results were compared with results generated by HAMMER and are shown to yield significant improvements in cortical alignment as well as reduced computation time.

## 1. Introduction

Image registration is a crucial tool in medical image analysis. Applications such as characterizing anatomical variability, monitoring changes due to disease or aging, and surgical planning have created the continuing need for research and development in registration [1–4]. The goal of medical image registration is to spatially align images so that they can be compared visually or quantitatively. In order for this alignment to be accurate, one must both identify correct anatomical correspondences between subjects and generate appropriate coordinate transformations that best align these correspondences. These are the central issues in deformable image registration and are the main focus of this paper.

 Deformable image registration has been approached in different ways (cf. [5–23]), and the algorithms can be divided into two main classes: intensity-based [5–9] and feature-based metheods [12, 17, 21–23]. Feature-based methods focus on aligning manually selected or automatically derived image landmarks. These methods are usually specialized for aligning same-modality images of a particular body part—for example, brain, breast, or heart. Automation is a key challenge since the algorithm must identify homologous landmarks or risk very large registration errors. Intensity-based methods, on the other hand, use dense subsets of image intensities to align images. The most popular and successful intensity-based methods to date use mutual information-based similarity criteria [9]. These algorithms are usually fast and fully automated and are very generic in that they are not limited to one particular body part or a single modality. However, the landmarks that are implied by these methods are not primarily based on anatomical homology, which can lead to erroneous deformations.

 A few methods that combine these two approaches have been proposed [10, 24–26]. HAMMER [10] combines intensity-based and feature-based registration by introducing two key concepts: attribute vectors and driving voxels. Information about both the image intensity and derived image features are concatenated into an attribute vector, which is defined for every voxel in both the template image and the subject image. Comparison of attribute vectors is analogous to the matching of image intensities. HAMMER's driving voxels are hierarchically selected voxels that represent strong, reliable features that are to be aligned first. This process reduces the number of suboptimal correspondences in the beginning of the registration process. The blending of both intensity-based and feature-based strategies puts HAMMER at the forefront of deformable registration algorithms today and is the main reason why we chose to use a similar framework for our algorithm.

 Despite its strengths, there are three components in HAMMER where significant improvements can be made. First, HAMMER computes its attribute vectors exclusively from a hard segmentation of the MR images. The hard segmentation is a strict classification of the image into three discrete classes, which correspond to the three main tissue types of the human brain, that is, the white matter (WM), gray matter (GM), and cerebrospinal fluid (CSF). Given HAMMER's attribute vectors and similarity criteria, this segmentation needs to be extremely consistent between subjects in order for the algorithm to be robust in identifying homologous anatomical landmarks. Second, because of HAMMER's exclusive use of a hard segmentation, it requires extra preprocessing of the images—a time consuming step—wherein the ventricles of the brain are segmented and used (essentially) as a fourth tissue class. Third, HAMMER's strategy to interpolate landmark correspondences in order to generate a dense displacement field is to apply nonoverlapping Gaussian kernels and use uniform smoothing throughout the entire image, that is, both on the highly convoluted brain cortex as well as in the more uniform structures of the subcortical white matter. This approach prevents the algorithm from capturing high-curvature and high-resolution details when registering the folding patterns—that is, sulci and gyri—of the brain cortex.

 In this paper, we address these three limitations of HAMMER in a new algorithm that we call Mjolnir. (The name choice was motivated by our goal to create a “better HAMMER”; Mjolnir was the name of the hammer of the Norse god Thor, arguably one of the most powerful hammers ever “made”.) We provide new strategies that both improve registration accuracy and reduce computation time. The main technical contribution of the paper is a novel, fast interpolation of the displacement field defined from sparse landmarks during the registration process (Section 4). We use a diffusion transformation model, which does not smooth the displacement field uniformly throughout the image but takes into account derived information about the quality of landmark correspondences and weights the smoothing based on the anatomical features being aligned. Other contributions include the incorporation of fuzzy classification and histogram equalization into the algorithm. Mjolnir was extensively evaluated using the NIREP (Non-Rigid Image Registration Evaluation Project [27, 28]) evaluation database and its performance compared to the publicly available HAMMER software [29]. Significant improvements are evident in nearly all comparative measures that are computed and are most strikingly evident in the cortex of the brain, which is a very difficult structure to register across subjects because of its high variability in the general population. The Mjolnir software will be made publicly available on http://iacl.ece.jhu.edu/ upon publication of this paper.

## 2. Overview of Major Concepts

**Figure 1:**
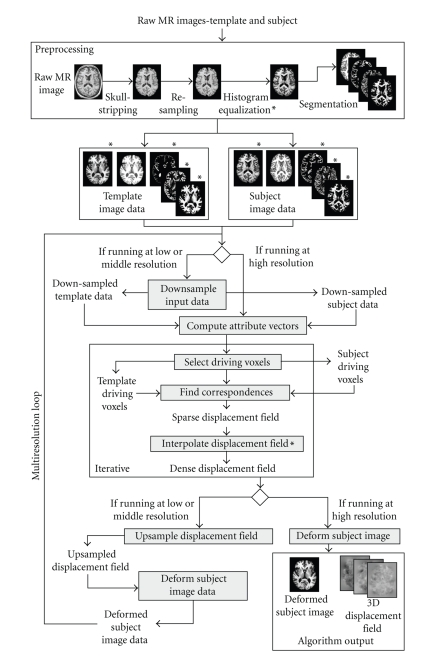
A flowchart demonstrating the major steps of Mjolnir. Elements of Mjolnir that are different from HAMMER are marked with *.

In this section, we focus on the two major areas of improvement that Mjolnir claims over HAMMER: ( 1) improved preprocessing procedures and ( 2) a novel transformation model that interpolates sparse landmarks in order to generate a dense displacement field. To provide context for these improvements, we first briefly describe Mjolnir's major processing concepts, which are graphically illustrated using the flowchart in Figure 1. Elements of Mjolnir that are different from HAMMER are marked with * in the figure. For further details about those processing steps that are in common with HAMMER, the reader is referred to the main HAMMER publication [10].

### 2.1. Attribute Vectors

Consider a voxel at position **x** in either the subject or the template image. An *attribute vector* **a**(**x**) is defined as the concatenation of specific regional image features that are computed within a small spherical neighborhood centered at **x**. There are three key parts of Mjolnir's attribute vector **a**(**x**) = (*a*_1_(**x**), *a*_2_(**x**), **a**_3_(**x**)), which are together used to measure image similarity during the registration process. The first part *a*_1_ is an integer that represents the *edge type* of the voxel based on a hard segmentation of the MR brain image into three classes, corresponding to the three main tissue types of the human brain: white matter (WM), gray matter (GM), and cerebrospinal fluid (CSF). The edge type takes one of seven values, one value for voxels that are not adjacent to an edge and six distinct values for voxels that are located on edges between WM, GM, and CSF.

 The second part *a*_2_ of Mjolnir's attribute vector is the intensity of a histogram-equalized version of the original MR image, normalized between zero and one over the image domain (see the rationale for histogram equalization in Section 3). A good registration will yield strong similarities in both edge type *a*_1_ and normalized intensity *a*_2_ when the images are aligned. The third part **a**_3_ of Mjolnir's attribute vector is a vector of normalized *geometric moment invariants* (GMIs) [30]. Several GMIs, which yield measures of the “intensity shape” around each voxel, are computed based on segmented MR images at a small neighborhood around each voxel of each image.

### 2.2. Driving Voxels

As in HAMMER, Mjolnir uses the concept of *driving voxels*. These are voxels with distinctive attribute vectors (in either image) that can be associated with similar points in the other image, creating temporary landmark pairs that “drive” the deformation of one image toward the other. Driving voxels are “active” points while all other voxels are “passive,” because the overall displacement field at any given iteration is determined entirely from the implied displacements of the driving voxels. 

 Driving voxels are automatically selected in a hierarchical fashion, starting with a small initial set of highly distinctive voxels in both the subject and template images. Voxels with attribute vectors that are similar—as measured by the similarity function defined in (1) below—to very few other places in the brain are deemed to be “highly distinctive.” The original HAMMER publication [10] demonstrated that voxels on sulcal fundi and gyral crowns are distinctive by this criterion. These *primary* driving voxels initialize the registration algorithm; in later iterations, the driving voxels in the template image are gradually augmented by adding an increasing number of (increasingly less) distinctive points in the neighborhood of each primary driving voxel. In each iteration, the subject image is deformed relative to the template image, and new driving voxels are identified in order to continue to bring the images into alignment. Various thresholds are changed throughout the registration process such that all voxels in the template image eventually become driving voxels. For computational savings, the driving voxels in the subject image are never augmented to include more than just the primary driving voxels.

### 2.3. Correspondences

For each driving voxel in the subject and template images, a corresponding voxel in the other image is found, creating a (temporary) landmark pair. This is performed by searching in the opposite image for matching voxels. The quality of a match is based on voxel similarity, which is defined for two voxels **x**_*s*_ and **x**_*t*_ using their attribute vectors as follows:

(1) $$s\left(x_{s},x_{t}\right)=\delta \left(a_{1}\left(x_{s}\right)-a_{1}\left(x_{t}\right)\right)\cdot \left(1-\left|a_{2}\left(x_{s}\right)-a_{2}\left(x_{t}\right)\right|\right)\cdot \overset{9}{\underset{i=1}{\prod }}\left(1-\left|a_{3}^{i}\left(x_{s}\right)-a_{3}^{i}\left(x_{t}\right)\right|\right);$$

where *δ*(·) is the discrete delta function. The range of similarity is [0, 1] where unity means perfect similarity. 

 After searching and finding correspondences for all driving voxels, displacement vectors pointing from the template to the subject are formed for each driving voxel, forming a sparse displacement field **u**_*c*_ in the domain of the template. This field must be interpolated and smoothed throughout the entire image domain in order to generate a dense displacement field (see Section 4), which yields a transformation that can be used to align the two images by warping their coordinate systems.

### 2.4. Overall Control Strategy

Mjolnir is run on three resolutions from coarse to fine (see Figure 1). By careful control of the parameters of the process—for example, size of search windows and growth rate of the number of driving voxels, (see Table 1)—the images are brought into alignment. As in HAMMER, the overall process can be thought of as one that combines landmark alignment through selection of the driving voxels with intensity-based alignment through use of attribute vector similarity. In the following sections we describe the parts of Mjolnir that have been substantially improved over HAMMER.

## 3. Preprocessing

Before running Mjolnir, a sequence of preprocessing steps must be applied to the raw MR images (see the top box in Figure 1). Mjolnir is specifically tuned for SPGR (SPoiled Gradient Recalled) T1-weighted MR images of the human brain with the following parameters: TE = 52 ms, TR = 35 ms, FOV = 24 cm, flip angle = 45 degrees, slice thickness = 1.5 mm, gap = 0, matrix = 256 × 256, NEX = 1. Use of alternate contrasts may require the use of a different classification algorithm and some changes to the attribute vector, the similarity measure, and thresholds on similarity. Once these changes are made, the rest of Mjolnir should work without modification.

 The preprocessing steps include skull-stripping, resampling, histogram equalization, and tissue classification (i.e., segmentation). Resampling yields cubic voxels, which reduces the directional dependency in subsequent processing. We do not discuss skull-stripping and resampling herein, as these are carried out in a conventional manner (cf. [31, 32]). However, our use of histogram equalization and the specific implementation and use of tissue classification is novel and important, so we briefly describe them below. Specific benefits resulting from these steps are shown in Section 5. 

**Figure 2:**
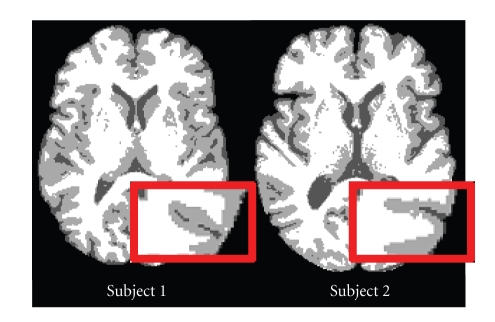
The hard segmentation of two different subjects.

 MR image intensities between subjects can be quite variable, even for the same pulse sequence and the same scanner. Differences may be caused by pulse sequence variations, intensity inhomogeneities, flow artifacts, scanner gain differences, and motion ghosting. This in turn can cause the hard segmentation of MR images to be very inconsistent between different scans. Inconsistencies between subjects are particularly evident on the brain cortex, as demonstrated in Figure 2. The highlighted sulci on these segmented images, which should be homologous and therefore registered to each other, are inconsistent in appearance; in particular, the CSF appears to be split in one subject but not the other. Because of this inconsistency, HAMMER's exclusive reliance on hard segmentations can cause registration errors. Mjolnir addresses this problem both by using histogram equalization and by incorporating the soft segmentations into the attribute vectors. 

### 3.1. Histogram Equalization

**Figure 3:**
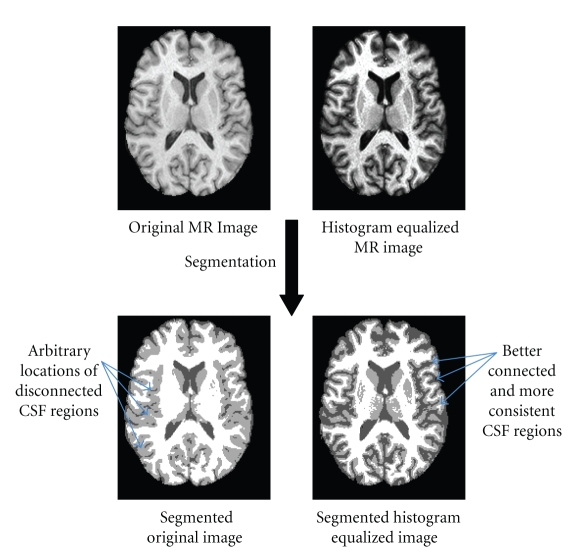
Demonstration of the effects of histogram equalization on the segmentation. The top row shows the original image without histogram equalization on the left and a histogram equalized image on the right. The bottom row shows the corresponding hard segmented images. The arrows show smoother edges and better connected regions of the CSF class when histogram equalization is applied before segmentation. Arbitrarily located CSF spots have been removed, thereby increasing the consistency of the CSF class between different subjects.

**Figure 4:**
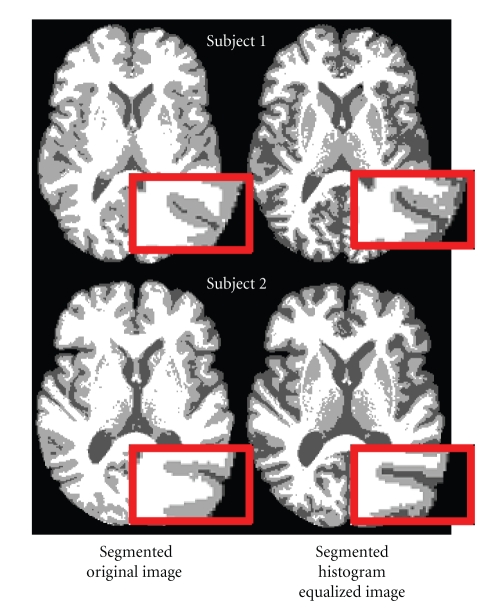
The figure shows the hard segmentation of two different subjects with and without histogram equalization before segmentation. This specific example shows how disconnected CSF spots become connected when applying histogram equalization before segmentation, thereby increasing the consistency of the CSF class between the two different subjects.

In Mjolnir, we use histogram equalization [33] of both images in order to create a more exact match between the intensities of the two images and to create sulcal features that are typically quite subtle in the original MR images. This procedure reduces differences and inconsistencies between different subject scans as well as increasing the intensity contrast between tissue classes, thereby improving their ability to define distinctive landmarks. In particular, we have noticed that histogram equalization yields hard segmentations that are more revealing of tissue class boundaries that are consistently defined across different subjects. This property is illustrated in Figure 3. In this example, regions of sulcal CSF are arbitrarily disconnected in the hard segmentation of the original image, whereas the CSF within sulci is largely connected when the histogram equalized image is used. This tends to increase the consistency of the CSF class between different subjects. A specific example on improved consistency between different subjects is shown in Figure 4. 

### 3.2. Geometric Moments

**Figure 5:**
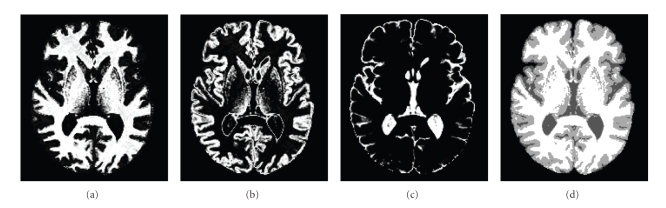
Fuzzy segmentation of an image into three membership functions. (a) the white matter (WM), (b) cgray matter (GM), (c) cerebrospinal fluid (CSF), and (d) Corresponding hard segmentation.

In both Mjolnir and HAMMER, skull-stripped and isotropically resampled images are classified into white matter (WM), gray matter (GM), and cerebrospinal fluid (CSF) using FANTASM [34, 35]. This algorithm compensates for intensity inhomogeneities while simultaneously generating three membership functions for WM, GM, and CSF (see Figures 5(a)–5(c)), representing a “fuzzy” or “soft” classification. A “hard” classification is created by assigning each voxel to the class having the highest membership value for that voxel. The segmentation result is used by both Mjolnir and HAMMER as the primary basis of their attribute vectors and therefore has a very significant influence on the accuracy of the final result. While HAMMER uses only the hard segmentation, Mjolnir uses the soft segmentation as well.

 Mjolnir computes geometric moments on the membership functions rather than the hard classifications as in HAMMER. In addition, Mjolnir incorporates the histogram equalized MR intensity values into its attribute vector whereas HAMMER uses the hard segmentation values instead. It is our observation that these two changes tend to yield more distinctive features throughout the brain, which provides better alignment during registration. We believe that these changes are the main reason why Mjolnir provides excellent alignment of the ventricles (see Section 5) without using a separate ventricle segmentation process as is done in HAMMER.

## 4. Displacement Field Interpolation

After preprocessing, driving voxels and their correspondences are found as previously described (see also flowchart in Figure 1). The resulting displacement vectors are generally sparse (except in the final iterations) and of varying reliability (as measured by their relative computed similarities (see (1)). In order to compute a dense coordinate transformation, these data must be interpolated throughout the entire image, and the stronger matches should maintain more influence on the resulting dense displacement field.

 HAMMER uses nonoverlapping Gaussian kernels applied to discrete subvolumes around each driving voxel in order to provide an initial dense displacement field. This field is then modified by both global and local affine transformations and then globally smoothed to remove discontinuities that are introduced on the boundary of each deformed subvolume. This process may oversmooth the displacement vectors corresponding to highly similar landmark voxels and may also leave evidence of the discontinuities at the edges of the supports of the Gaussian kernels.

 Numerous interpolation methods have been proposed for interpolation of scattered data. Some have been used in deformable image registration [11]; however, literature is not so abundant when it comes to the interpolation of vector fields, which is required in our case. Rueckert et al. [5] used a free form deformation (FFD) model based on B-splines that deforms the image by manipulating an underlying mesh of uniformly spaced control points. A key advantage of B-splines is that they have local control such that changing a control point affects the transformation only in the local neighborhood of that point. This also makes B-splines computationally efficient; however, large numbers of control points are required in order to capture the fine details of cortical registration, and this greatly increases the computational complexity. Thin-plate splines have also been used to provide coordinate system transformations in registration [12]. Since these are not local models, computational complexity is prohibitive for all but relatively low-order (smooth) transformations. Kriging is another widely used technique for spatial interpolation from sparse data [36], and although local neighborhood kriging has been proposed, it remains computationally prohibitive for large numbers of observed points. Mjolnir's strategy starts with a relatively sparse interpolation problem, and for this phase the above approaches could be used. But as Mjolnir concludes its registration process, the numbers of observed displacements eventually equals the total number of voxels in the image volume, which is not computationally feasible for these existing interpolation and smoothing algorithms. We clearly need a computationally feasible interpolation strategy that will work for vector fields, provides a smoothed interpolation (not an exact fit), and works for both scattered and dense observations.

 Mjolnir uses a novel diffusion model to interpolate the displacement field. It features all the properties mentioned above, in addition to providing for variable weights based on reliability. Consider the sparse displacement field **u**_*c*_ computed by matching driving voxels. The first requirement for the dense displacement field **u** is that it follows the displacement vectors of the sparse displacement field **u**_*c*_ that were derived from the landmark pairs, since these displacement vectors represent correspondences that have been carefully evaluated. The second requirement is that the displacement field is smooth across the entire image. In order to satisfy these requirements, we define the dense displacement field to be the vector field **u**(**x**) that minimizes the following energy functional;

(2) $$E\left(u\left(x\right)\right)=\frac{1}{2}\int \;g{\left|\nabla u\left(x\right)\right|}^{2}+p\left(x\right){\left|u\left(x\right)-u_{c}\left(x\right)\right|}^{2}dx,$$

where ∇ is the gradient operator. The first term in the integrand of (2) encourages a smooth displacement field while the second term encourages the resultant field to agree with the established landmark displacement vectors. We now describe the selection of the two weights, *g* and *p*(**x**).

 The computed displacement field should agree with the landmark displacement vectors when they are accurate. Accordingly, *p*(**x**) is defined as

(3) $$\begin{array}{ll} p\left(x_{t}\right) & =\{\begin{array}{cc} 2s\left(x_{s},x_{t}\right), & \text{if}\;x_{t}\;\text{is}\;\text{a}\;\text{template}\;\text{driving}\;\text{voxel}\;\\ & \text{located}\;\text{on}\;\text{an}\;\text{edge,}\\ s\left(x_{s},x_{t}\right), & \text{if}\;x_{t}\;\text{is}\;\text{a}\;\text{template}\;\text{driving}\;\text{voxel}\\ & \text{not}\;\text{located}\;\text{on}\;\text{an}\;\text{edge,}\\ 0, & \text{otherwise,} \end{array} \end{array}$$

where *s*(**x**_*s*_, **x**_*t*_) is the similarity function defined in (1). With this definition, landmarks that are very similar—that is, *s* ≈ 1—are given more weight than nonlandmarks and landmarks with lower similarity. Those that are located on edges are given twice the weight since it is highly desirable to align edges.

**Figure 6:**
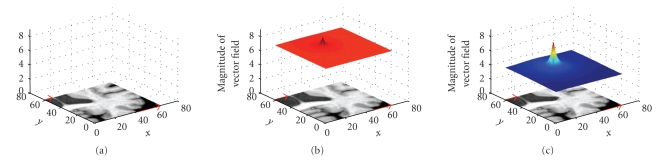
2D demonstration of the vector field interpolation around a small cluster of displacement vectors. (a) Image before. (b) Deformed image and corresponding displacement field (magnitude) with high smoothing, hence a large influence zone. (d) Deformed image and corresponding displacement field (magnitude) with low smoothing, which leads to more localized movement.

 The spatially constant weight *g* in (2) controls the tradeoff between smoothness and data fidelity. It is set to be large in the beginning of the registration process when **u**_*c*_ is sparse and then to become smaller in later iterations. In this way, the spatial influence of each landmark is large in the beginning and becomes smaller as the density of driving voxels increases. The effects of the regularization parameter *g* are demonstrated in 2D in Figure 6.

 This approach to displacement vector interpolation provides a more principled interpolation than the approach of HAMMER. The displacement field will be smooth wherever there are few strong features, such as white matter regions, yet will enable intricate displacement fields where there are many strong features, such as the gray matter cortex. Both qualitative and quantitative benefits of using this displacement field interpolation over that used in HAMMER are demonstrated in Section 5.1.

 The Euler-Lagrange equation corresponding to (2) is given by

(4) $$g\nabla^{2}u-p\left(u-u_{c}\right)=0.$$

We solve this equation using a fast and efficient multigrid approach [37] as described in [38] (which solves this same equation for the computation of gradient vector flow (cf. [39, 40])). This equation can also be solved by gradient descent, leading to the gradient flow

(5) $$u_{t}=g\nabla^{2}u-p\left(u-u_{c}\right),$$

whose equilibrium state is the desired solution.

 The iterative process shown in the bold box of Figure 1 is deemed to be complete when either the maximum difference between the current computed dense displacement field **u** ^(*k*)^ and that of the previous iteration **u** ^(*k*−1)^ differs in length by less than one voxel or when a preset *maximum number of iterations* has been achieved. Upon completion of this iterative process at low resolution, the iterative process is repeated at medium and then high resolutions, upsampling and deforming the subject data between each resolution. Mjolnir finishes by deforming the subject image using the dense field computed upon convergence of the iterative process at the highest resolution (see Figure 1).

## 5. Results and Discussion

In the past, evaluating the performance of deformable registration algorithms has been accomplished through use of simulated deformation fields, demonstration of average images in atlas space, and comparison with small numbers of manually selected landmarks [41–45]. With the advent of the Non-Rigid Image Registration Evaluation Project (NIREP) [27, 28], it is now possible to additionally evaluate the performance of a deformable registration algorithm through its ability to transfer labels between images. The NIREP Na0 database comprises 16 richly annotated 3D T1-weighted SPGR MR brain images corresponding to eight males and eight females, all normal adults. The 16 datasets have been manually segmented into 32 gray matter regions of interest, including the frontal, parietal, temporal, and occipital lobes, the cingulate gyrus and insula [28].

 In particular, both Mjolnir and HAMMER were used to register 15 of the NIREP subjects to a randomly selected “template image”, and the deformed labels were then compared to the corresponding labels of the chosen template. The same image was used as a template in all our experiments. The average Dice coefficient for each template label and for each algorithm was then computed, where Dice coefficient is defined as [46]

(6) $$D\left(S_{1},S_{2}\right)=\frac{2\left|S_{1}\cap S_{2}\right|}{\left(\left|S_{1}\right|+\left|S_{2}\right|\right)},$$

where *S*_1_ and *S*_2_ are two labeled regions, and |*S*| is the number of voxels in *S*. This measure gives a quantitative evaluation of the algorithm's performance in aligning anatomical structures. In our analysis of the overlap measure we used the paired *t*-test [47] to determine if the difference in average Dice coefficient between Mjolnir and HAMMER was statistically significant. We did the hypothesis testing on each region's difference of the mean in the following way. The average Dice coefficient for each of the 32 cortical regions for both algorithms was computed, yielding 32 pairs of data (*X*_*i*_, *Y*_*i*_), *i* = 1,…, 32, where *X*_*i*_ is the average Dice coefficient for Mjolnir, and *Y*_*i*_ is the average Dice coefficient for HAMMER. Since each of the cortical regions are inherently different, we cannot treat *X*_1_,…, *X*_32_ and *Y*_1_,…, *Y*_32_ as being independent samples. The hypothesis testing was therefore applied on each region's difference of the means. We constructed *W*_*i*_ = *X*_*i*_ − *Y*_*i*_, *i* = 1,…, 32 and tested the hypothesis of no improvements in registration accuracy by testing *H*_0_ : *x* − *y* = 0 versus *H*_1_ : *x* − *y* ≠ 0, where *H*_0_ is our null hypothesis. If the *P*-value for the null hypothesis is less than .05, our hypothesis that there is no difference in average Dice coefficient between Mjolnir and HAMMER is rejected; hence, we can conclude that the difference in Dice coefficient is statistically significant.

In the following experiments we compared the performance of Mjolnir with the publicly available HAMMER program [29]: HAMMER Version1.0 @ SBIA. Our aim was to demonstrate the benefits of our extensions to the HAMMER algorithm and their effects on registration accuracy, and the experiments were designed with that in mind. All the experiments were performed by us using the default parameter values of HAMMER, and we chose the same parameter values for Mjolnir (see Table 1). The performance of the two algorithms was tested on the NIREP evaluation database http://www.nirep.org/, which, at the time of testing, was “unseen” data to both of the algorithms to prevent any bias of favoring one algorithm over the other; that is Mjolnir was not specifically “tuned” for this dataset in any way prior to carrying out the analyses presented below.

### 5.1. Specific Benefits of Mjolnir

The efficacy of the three major differences between Mjolnir and HAMMER was evaluated using the NIREP data.

#### 5.1.1. Histogram Equalization

**Figure 7:**
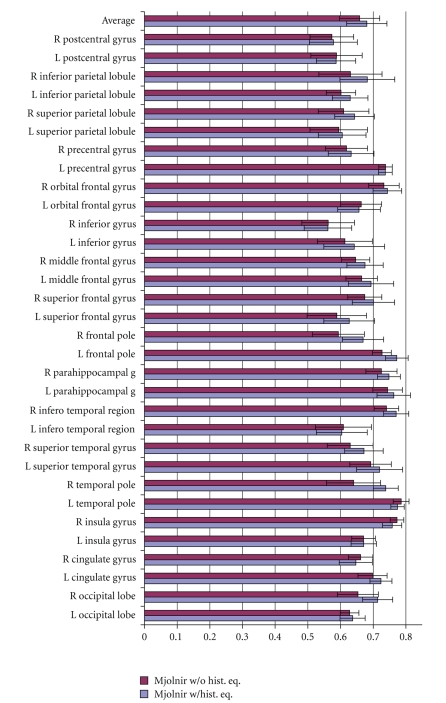
Average Dice coefficients for different anatomical regions on the left and right hemisphere (labeled L and R) for Mjolnir registration with and without histogram equalization in its preprocessing routine. The top set of bars shows the average over all regions and the error bars represent one standard deviation in each direction.

We ran Mjolnir with and without histogram equalization first to measure directly the benefits of including histogram equalization in the preprocessing routine. All other parameters were kept the same. We registered 15 MR brain images to a 16th randomly selected template image. The average Dice coefficient was computed to measure the overlap between corresponding regions of all subjects after registration. Results are shown in Figure 7.

 The registration accuracy was improved in 25 out of the 32 labeled regions of the brain when using histogram equalization first, as shown in Figure 7. The top row of the bar chart shows the mean value over all regions. The mean value was 0.6584 with standard deviation (SD) of 0.0614 when Mjolnir was run without the histogram equalization first and 0.6804 with SD of 0.0617 when histogram equalization was included in the preprocessing routine. Statistical analysis of these results using the paired *t*-test indicate that this difference is statistically significant (*P* = 4.04 × 10^−05^).

#### 5.1.2. Soft Segmentation versus Hard Segmentation

**Figure 8:**
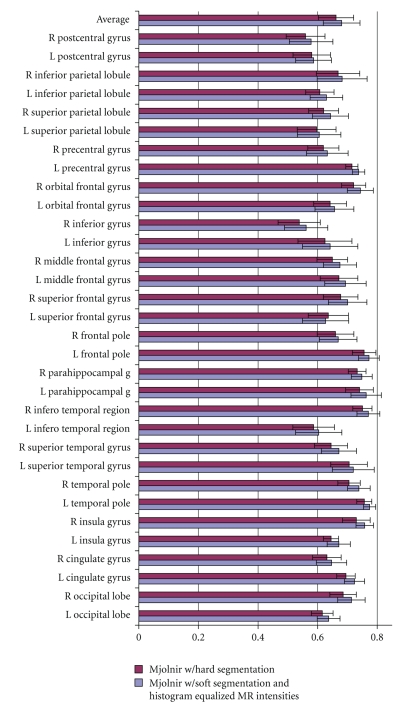
Average Dice coefficients for different anatomical regions on the left and right hemisphere (labeled L and R) for Mjolnir registration using hard segmentation exclusively (as in HAMMER) versus using fuzzy (or soft) segmentation and histogram equalized MR intensities in the attribute vector. The top set of bars shows the average over all regions, and the error bars represent one standard deviation in each direction.

We modified Mjolnir to use only a hard segmentation in its attribute vector (as in HAMMER) so that a measure of the benefit of using a soft segmentation and histogram equalized MR intensities could be evaluated directly. Histogram equalization was used in the preprocessing steps to make sure that the edge type element *a*_1_(**x**) in the attribute vector was the same in both versions of Mjolnir used in this experiment. All other parameters were kept the same. We registered 15 MR brain images to a 16th randomly selected template image. The average Dice coefficient was computed to measure the overlap between corresponding regions of all subjects after registration. Results are shown in Figure 8. 

Figure 8 reveals that registration accuracy is improved in all regions of the brain. The top row of the bar chart shows the mean value over all regions. The mean value was 0.6615 with SD of 0.0593 when using HAMMER's attribute vector and 0.6804 with SD of 0.0617 when using Mjolnir's attribute vector. Statistical analysis of these results using the paired *t*-test indicate that significant improvements in registration accuracy of all cortical regions were seen when using Mjolnir's full attribute vector (*P* = 2.94 × 10^−14^).

#### 5.1.3. Displacement Field Interpolation

**Figure 9:**
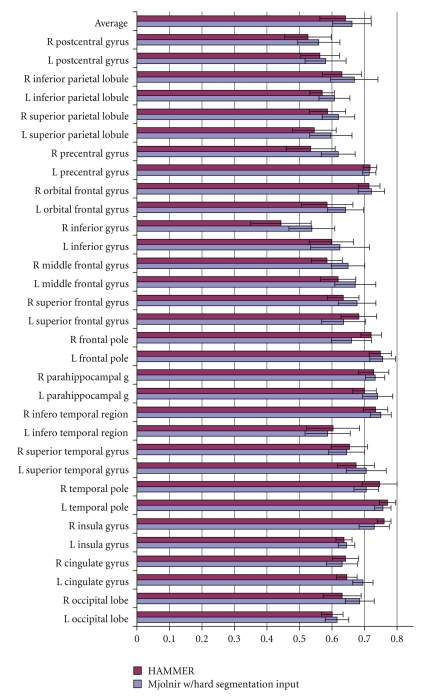
Demonstration of the benefits of Mjolnir's displacement field interpolation. The input to both algorithms (HAMMER and Mjolnir) was the hard segmentation of the image; hence the attribute vectors in both methods are the same. The top row shows the average over all regions, and the error bars represent the standard deviation.

To demonstrate the benefits of Mjolnir's displacement field interpolation, we modified Mjolnir to use HAMMER's attribute vector in the following way: ( 1) we used the hard segmentation instead of the histogram equalized MR intensity for the intensity element *a*_2_(**x**), and ( 2) we computed the GMI's in **a**_3_(**x**) based on the hard segmentation instead of the soft segmentation. Therefore, the dominant difference between this modified version of Mjolnir and HAMMER was the displacement field interpolation strategy, although the two algorithms differed in their preprocessing steps as well. As in the previous experiment, we registered 15 subjects to a 16th template image; results are shown in Figure 9. 

It is observed from Figure 9 that Mjolnir's interpolation strategy yields better alignment in 23 out of 32 structures. The average Dice coefficient over all regions (top row) is larger in Mjolnir, measuring 0.6416 with SD of 0.0789 when using HAMMER's interpolation approach and 0.6615 with SD of 0.0325 when using Mjolnir's approach. The results of a statistical analysis using a paired *t*-test demonstrate a significant improvement of Mjolnir's interpolation strategy over that of HAMMER (*P* = .0046).

### 5.2. Algorithm Comparison

#### 5.2.1. Dice Coefficients on Labeled Regions

**Figure 10:**
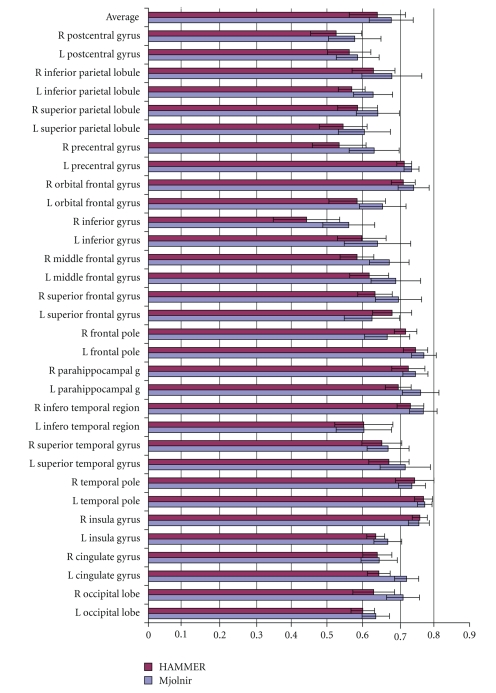
Average Dice coefficient for different anatomical regions on the left and right hemisphere (labeled L and R) for HAMMER and Mjolnir. The top set of bars shows the average over all regions, and the error bars represent the standard deviation.

We evaluated the two registration algorithms using the NIREP database by choosing one brain randomly as a template, registering the 15 remaining brains to this template and computing the average Dice coefficient, region-by-region and overall. The results are shown in Figure 10. It is observed that Mjolnir gives a better alignment in 28 out of 32 of the labeled cortical structures than does HAMMER and gives a higher average Dice coefficient across all regions of the brain (top row in Figure 10), measuring 0.6804 with an SD of 0.0617 while HAMMER's average Dice coefficient was 0.6416 with an SD of 0.0789. In a statistical analysis using a paired *t*-test this result is shown to be statistically significant with a *P*-value of 4.02 × 10^−06^. 

 In order to determine whether the choice of template might bias the result, we repeated the above experiment using a different randomly selected template image. Mjolnir gave again a better alignment in 29 out of 32 labeled cortical structures. It also gave a significantly higher average Dice coefficient than HAMMER (*P* = 3.71 × 10^−07^), measuring 0.6507 with an SD of 0.0759 for HAMMER and 0.6866 with an SD of 0.0632 for Mjolnir. 

**Figure 11:**
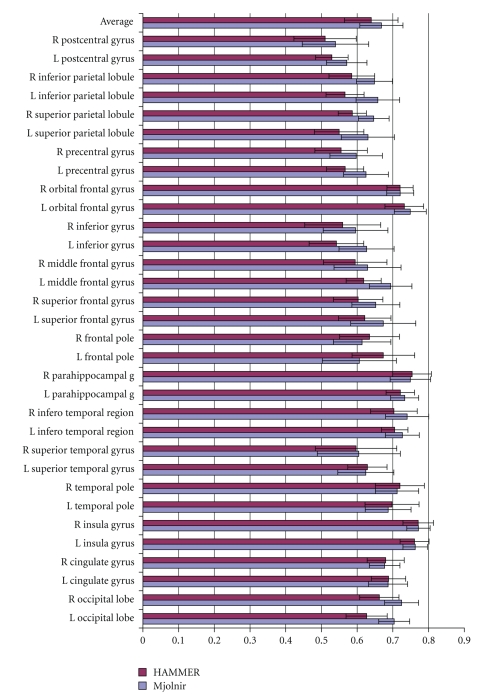
Average Dice coefficient for different anatomical regions on the left and right hemisphere (labeled L and R) for HAMMER and Mjolnir when registering 16 randomly selected image pairs. The top set of bars shows the average over all regions, and the error bars represent the standard deviation.

 Finally, in order to eliminate any bias from registering all subjects to a single template image we randomly selected 16 image pairs (with one randomly selected as the template). The average Dice coefficients for the 16 pairwise registrations were computed and are shown in Figure 11. It is observed that Mjolnir gives a better alignment in 24 out of 32 of the labeled cortical structures than does HAMMER and also gives a higher average Dice coefficient across all regions of the brain, measuring 0.6680 with an SD of 0.0604 while HAMMER's average Dice coefficient was 0.6394 with an SD of 0.0748. In a statistical analysis using a paired *t*-test this result is shown to be statistically significant with a *P*-value of 8.07 × 10^−05^.

#### 5.2.2. Average Images

A common method used to measure image registration performance is to register multiple subjects to one template image and then compute the average of the registered images [6, 10, 27, 41] as follows:

(7) $$\overset{̅}{I}\left(x\right)=\frac{1}{N}\overset{N}{\underset{i=1}{\sum }}I_{i}\left(x+u_{i}\left(x\right)\right).$$

Here, *I*_*i*_ is the *i*th image in the population, and **u**_*i*_(**x**) is the displacement field from subject *i* to the template. The best registration approach yields the least noisy and “sharpest” appearing average image, which can be quantitatively assessed by computing the variance of the registered image intensities [27] as follows:

(8) $$\sigma_{I}^{2}\left(x\right)=\frac{1}{N-1}\overset{N}{\underset{i=1}{\sum }}{\left(I_{i}\left(x+u_{i}\left(x\right)\right)-\overset{̅}{I}\left(x\right)\right)}^{2}\;.$$

Small variances throughout the field of view indicates a good registration of all subjects to the template; the average variance across all pixels provides a single figure of merit for algorithm comparison.

**Figure 12:**
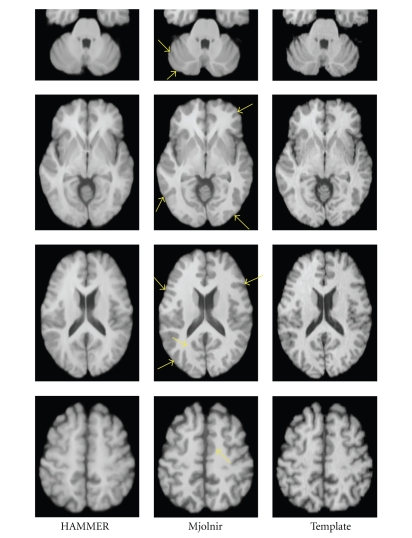
Four cross sections of the averages of 15 images registered to a 16th template image. The arrows highlight the most apparent regions of improvement in Mjolnir over HAMMER.

 As in previous experiments, we registered 15 subjects to a randomly selected template, using all images in the NIREP Na0 database. Average and variance images were computed for both Mjolnir and HAMMER. Figure 12 shows a cross-sectional view of the two average images together with the template image. It can be observed that the average image generated by Mjolnir is sharper than that of HAMMER, particularly within the cortex. The average image variance over all voxels within the template's brain was 52.3 for HAMMER and 34.5 for Mjolnir (where the intensity range of the images was (0, 255)). 

#### 5.2.3. Cortical Gray Matter Alignment

Even when the images are correctly aligned, some variations in intensities between subjects can be expected simply due to both the lack of MR image intensity calibration and the natural variations in the MR properties of human tissues. In this experiment, we explore whether gray matter is well aligned by the two registration algorithms, despite possible underlying intensity variations. In order to visualize this effect, gray matter masks were generated from the labeled “subject” images in the NIREP Na0 database as follows:

(9) $$M_{i}\left(x\right)=\{\begin{array}{cc} 1, & \text{if}\;x\;\text{is}\;\text{labeled}\;\text{as}\;GM\;\text{in NIREP,}\\ 0, & \text{otherwise,} \end{array}$$

for *i* = 1,…, 15 (the “subject” images). After registration of each subject to the template, the sum of the deformed masks was formed as follows:

(10) $$M\left(x\right)=\overset{15}{\underset{i=1}{\sum }}M_{i}\left(x-u_{i}\left(x\right)\right).$$

**Figure 13:**
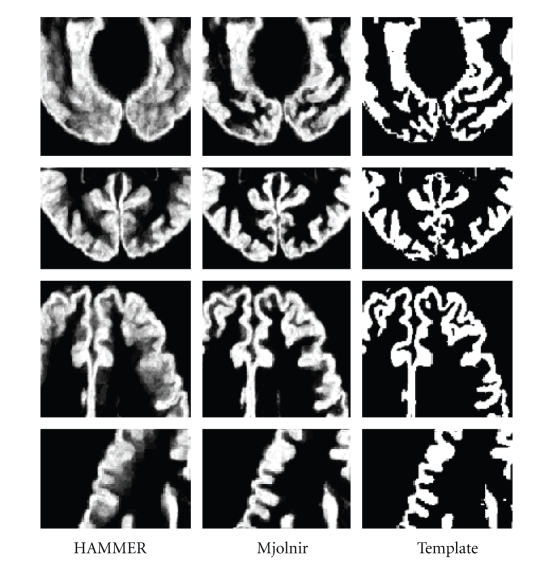
The distribution of the aligned gray matter. The gray matter of the template is shown on the right column for comparison. Evidences of large outlier errors are visible in the result generated by HAMMER (see blurring of boundaries).

**Figure 14:**
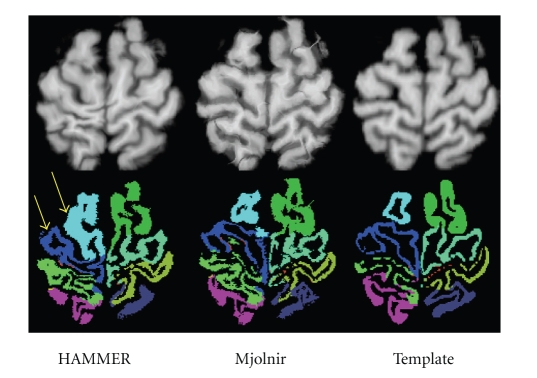
Example of an outlier occurring in HAMMER, where HAMMER's ability to follow the folding pattern of the cortex is lacking. The top row shows the deformed MR images and the corresponding template image on the right. The bottom row shows the corresponding deformed labels from the NIREP Na0 database and the template's labels.

Figure 13 shows the sum of deformed mask points for both algorithms in the first two columns and the original template gray matter mask on the right for comparison. Visually, it is clear that Mjolnir yields fewer gross errors—that is, outliers—than does HAMMER. Figure 14 shows an example of the (labeled) gray matter mapping results from both HAMMER and Mjolnir, both in comparison to the true template labels. This example demonstrates a rather large alignment error in the HAMMER result, while the Mjolnir result is overall more accurate. While Mjolnir is not perfect, the more compact GM mask alignment revealed in Figure 13 together with the improvements in average Dice coefficient shows that these types of gross errors are less common in Mjolnir than in HAMMER. Some evidences of tissue shearing are visible in the results of Mjolnir shown in Figure 14. The fact is that dramatic deformations must sometimes take place in order to best align homologous brain structures. This is particularly true when aligning different brains. Most of Mjolnir's deformation fields are fairly smooth; however, when a dramatic change is required in order to align important landmarks, it has the flexibility to do so. Figure 14 is an example of such a case. 

**Figure 15:**
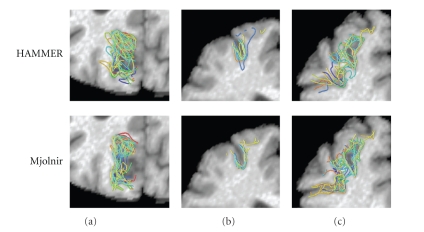
A total of 18 images were registered to a 19th template image. Manually labeled sulci on each of the subjects were deformed and superimposed to see the distribution after registration using HAMMER (top row) and Mjolnir (bottom row). (a) Parieto-occipital sulcus, (b) Superior frontal sulcus (view 1), and (c) Superior frontal sulcus (view 2).

 To further investigate the nature of outliers, we examined the alignment of specific sulci using the two methods. We used CRUISE [35] to extract central cortical surfaces from T1-weighted SPGR volumetric MR data set obtained from the Baltimore Longitudinal Study of Aging (BLSA) [1]. Sulcal regions—for example, cortex surrounding major sulci—were then segmented using the method of Rettmann et al. [48] and manually labeled on all 19 brain images. We then registered a total of 18 subjects to a randomly selected 19th template image using Mjolnir and HAMMER, and the extracted sulcal regions were then deformed using displacement fields derived from the registration processes. Figure 15 shows the 18 superimposed sulcal regions for the left parieto-occipital and the left superior frontal sulcus (two different views). The sulcal alignment in Mjolnir is more compact, while HAMMER produces a more spread alignment with larger registration errors, which is consistent with previous results from the NIREP data.

#### 5.2.4. Runtime

The two programs, Mjolnir and HAMMER (Version1.0 @ SBIA with default parameter values), were run on the same machine (Pentium Xeon 3.0 GHz, 4 GB RAM), on the same dataset (image size 256 × 256 × 198 voxels), and included all required preprocessing steps. HAMMER finished in 3 hours and 22 minutes while Mjolnir finished in 2 hours and 50 minutes, a 15% decrease in runtime.

## 6. Conclusion

Mjolnir, a new deformable registration algorithm introduced in this paper, has several significant benefits over HAMMER, the algorithm upon which Mjolnir was based. Among these are improvements to image preprocessing, more descriptive attribute vectors, improved vector field interpolation, and reduced computation time. Mjolnir was extensively validated both qualitatively and quantitatively using the NIREP Na0 evaluation database, and its performance was compared with the publicly available HAMMER. Results showed significant improvements in terms of overlap measure using the Dice coefficient of 32 registered gray matter structures of the brain cortex. As well, the intensity variance of 15 coregistered images was lower for Mjolnir as compared to HAMMER. By several additional measures, Mjolnir has fewer registration outliers than does HAMMER. Finally, Mjolnir showed a 15% decrease in runtime. 

 Most of Mjolnir's improvements were observed in the brain cortex, which has high variability in the general population (as compared to subcortical structures) and is critically important in the study of populations for analysis of regional shape, thickness, and volume changes due to aging and disease [1, 49, 50]. It is therefore hoped that Mjolnir might enable more compact geometric clustering of observed differences and/or to have a larger effect size, permitting smaller studies to observe statistically significant results.

## Figures and Tables

**Table 1:** Parameters used in Mjolnir in the following experiments.

	Resolution		
Parameters	Low	Mid	High
*R*_*G*_ (voxels): radius of spherical neighborhood in GMI computations	3	3	7
*D* (voxels): defines the size of the search region for finding correspondences (see *R*_*s*_ below)	12	10	8

maxIter: the maximum number of iterations		maxIter = 50	
iter ∈ (0 : maxIter): the current iteration			
*α* = iter/maxIter			
*R*_*s*_ (voxels): radius of spherical search neighborhood	*R*_*s*_ = 0.5*D* *e* ^*α*^2^/0.32^ + 1		
*t*_vox_: similarity threshold for voxel similarity	*t*_vox_ = 0.8(1 − *α*) + 0.01		
*t*_vol_: similarity threshold for average volume similarity	*t*_vol_ = 0.6(1 − *α*) + 0.01		
*σ*: the variance of the Gaussian kernel	*σ* = *R*_*s*_/3		
*w*1: weight in displacement field	*w*1 = 0.7		
*w*2: weight in displacement field	*w*2 = 0.3		
*g*: smoothing control in interpolation PDE	*g* = 0.8 − (0.8 − 0.45)*α*		
